# SAE1 promotes human glioma progression through activating AKT SUMOylation-mediated signaling pathways

**DOI:** 10.1186/s12964-019-0392-9

**Published:** 2019-07-25

**Authors:** Yanfang Yang, Ziwei Liang, Zijing Xia, Xixi Wang, Yanni Ma, Zenghua Sheng, Qingjia Gu, Guobo Shen, Liangxue Zhou, Hongxia Zhu, Ningzhi Xu, Shufang Liang

**Affiliations:** 1 State Key Laboratory of Biotherapy and Cancer Center, West China Hospital, Sichuan University, and Collaborative Innovation Center for Biotherapy, No.17, 3rd Section of People’s South Road, Chengdu, 610041 People’s Republic of China; 2 Department of Rheumatology and Immunology, West China Hospital, Sichuan University, Chengdu, 610041 Sichuan People’s Republic of China; 3 Department of Otorhinolaryngology, University of Electronic Science and Technology of China, Sichuan Academy of Medical Sciences & Sichuan Provincial People’s Hospital, Chengdu, China; 4 Department of Neurosurgery, West China Hospital, Sichuan University, Chengdu, 610041 Sichuan People’s Republic of China; 5 Laboratory of Cell and Molecular Biology & State Key Laboratory of Molecular Oncology, Cancer Institute & Cancer Hospital, Chinese Academy of Medical Sciences, Beijing, 100034 People’s Republic of China

**Keywords:** Glioma, SAE1, SUMOylation, Phosphorylation, Akt signaling pathway

## Abstract

**Background:**

The SUMO-activating enzyme SAE1 is indispensable for protein SUMOylation. A dysregulation of SAE1 expression involves in progression of several human cancers. However, its biological roles of SAE1 in glioma are unclear by now.

**Methods:**

The differential proteome between human glioma tissues and para-cancerous brain tissues were identified by LC-MS/MS. SAE1 expression was further assessed by immunohistochemistry. The patient overall survival versus SAE1 expression level was evaluated by Kaplan–Meier method. The glioma cell growth and migration were evaluated under SAE1 overexpression or inhibition by the CCK8, transwell assay and wound healing analysis. The SUMO1 modified target proteins were enriched from total cellular or tissue proteins by incubation with the anti-SUMO1 antibody on protein-A beads overnight, then the SUMOylated proteins were detected by Western blot. Cell apoptosis and cell cycle were analyzed by flow cytometry. The nude mouse xenograft was determined glioma growth and tumorigenicity in vivo.

**Results:**

SAE1 is identified to increase in glioma tissues by a quantitative proteomic dissection, and SAE1 upregulation indicates a high level of tumor malignancy grade and a poor overall survival for glioma patients. SAE1 overexpression induces an increase of the SUMOylation and Ser473 phosphorylation of AKT, which promotes glioma cell growth in vitro and in nude mouse tumor model. On the contrary, SAE1 silence induces an obvious suppression of the SUMOylation and Ser473 phosphorylation of Akt, which inhibits glioma cell proliferation and the tumor xenograft growth through inducing cell cycle arrest at G2 phase and cell apoptosis driven by serial biochemical molecular events.

**Conclusion:**

SAE1 promotes glioma cancer progression via enhancing Akt SUMOylation-mediated signaling pathway, which indicates targeting SUMOylation is a promising therapeutic strategy for human glioma.

**Electronic supplementary material:**

The online version of this article (10.1186/s12964-019-0392-9) contains supplementary material, which is available to authorized users.

## Background

Brain tumor is one of main cancers with increasing mortality among children and adults [1]. As a fatal primary malignant tumor, glioma is always associated with a poor prognosis along with high morbidity and mortality [2, 3]. Recently new molecular mechanism clarification for glioma provides a valuable insight into underlying biological features of disease [3, 4]. Protein SUMOylation, one post-translational modification (PTM), plays essential roles in various biological functions, which tightly relates with tumorigenesis [5, 6] and glioma development [7, 8].

SUMOylation is the small ubiquitin-like modifier (SUMO)-dependent biological modification process with the form of conjugation of SUMO molecule to an acceptor lysine of target proteins. SUMO modification is performed by three enzymatic cascade steps, including the heterodimer E1 enzyme (SAE1 and SAE2/UBA2)-involved activation, the E2 enzyme Ubc9-mediated conjugation and substrate modification through the cooperation of the E2, E3 protein ligases [6, 9].

SUMOylation is one important physiological mechanism in cellular responses to stress that is usually abnormal in many cancers [6, 10]. Enzymes relevant to SUMO conjugation pathway are dysfunctional in human disease states, which break a balance between the SUMOylation level with the modified cellular substrates and thereby leading to tumorigenesis. For instance, SUMO E3 protein PIAS3 is up-regulated in a number of different cancer types, such as lung, breast, prostate, colon cancer and brain tumor [11]. The Ubc9, a sole SUMO E2 in SUMO system, and global SUMOylated substrate proteins are markedly elevated in glioblastoma [7]. So far, protein SUMOylation pathway could be a new target of therapeutic intervention, and small-molecule inhibitors targeting SUMOylation modification are promising to develop into novel anticancer drugs [7, 12, 13].

Quantitative proteomics is powerful for identification of novel cancer protein markers [14–16] and protein SUMOylation [9, 17]. Our previous work has discovered each member of 14–3-3 isoforms and heat shock proteins has different expression level and biological function in human glioma by the stable isotope labeling with amino acids in cell culture (SILAC)-based proteomics dissection [18, 19]. In present study, we systematically identify differential expression proteome between human glioma tissues and its para-cancerous counterparts, from which a marked upregulation protein SAE1 is specially uncovered SAE1 roles in Akt SUMOylation-activated glioma development in vitro and in vivo.

## Materials and methods

### Cell culture

Human glioma cell line U87 was ordered from American Type Culture Collection (Manassas, VA), and U251 was ordered from the Type Culture Collection of the Chinese Academy of Sciences (Shanghai, China). Cells were cultured in DMEM medium (Hyclone) supplemented with 10% fetal bovine serum (FBS) [4]. The cell lines were authenticated by short tandem repeat analysis. In protein quantitative identification by mass spectrometry (MS), cells were cultured in deuterated-leucine (Leu-d_3_) (5, 5, 5-D_3_, 98%; Cabridge Isotope Laboratories, UK) containing DMEM medium with 10% dialyzed FBS (16000–044, Gibco, USA) until SILAC labeling was up to 95% [18–20].

### Glioma tissues

Sixty nine cases of human glioma tissues (HGTs) and twelve para-cancerous brain tissues (PBTs) were included in the study, which were endowed from glioma patients suffering from surgical resection in West China Hospital, Sichuan University of China with informed consents [4]. This study was approved by the Institutional Ethics Committee of State Key Laboratory of Biotherapy, West China Hospital of Sichuan University. The histological diagnosis was identified from the morphology and immunohistochemistry analysis. All tissue samples were obtained immediately after surgical resection and stored frozen with liquid nitrogen. The patient follow-up was performed mainly as our previous report [4]. The pathological grade of glioma tissues was assessed according to the tissue structure and cell characteristics under the microscopy observation by two medical doctors.

### Immunohistochemistry

SAE1 expression levels between HGTs and PBTs were semi-quantitatively compared by immunohistochemistry (IHC) scoring [21, 22]. Tissue sections were incubated with SAE1 antibody (ab56957, Abcam) at 4 °C overnight followed incubating with horseradish peroxidase-linked secondary antibodies at 37 °C for 40 min, then reacting with 3,3′-diaminobenzidine substrate solution (Dako Cytomation GmbH) and counterstaining with hematoxylin. Three independent fields at 20-fold magnification for positive cells were chosen to evaluate the immunostaining intensity and percentage. The immunoreactivity scores were measured the sum of immunostaining intensity multiplied by percentage of positive cells following our previous approaches [4, 22, 23]. The immunoreactivity score of SAE1 less than 4 was defined a low expression level, and the score more than 4 was defined a high expression of SAE1.

### Association analysis of SAE1 level with glioma clinical information

The association of SAE1 expression with glioma clinical information was assessed through the IHC scoring data of HGTs and PBTs using Pearson’s χ^2^ test [4]. The glioma patient’s gender, age and tumor grades were included to analyze. According to the SAE1 expression level, 69 human glioma patients were classified into two groups, low (Low, *n* = 21) and high SAE1 expression (High, *n* = 48). The patient overall survival was evaluated by Kaplan–Meier method. And a log-rank test was used to determine the statistical significance.

### MS identification

The differential proteome between HGTs and PBTs were identified by LC-MS/MS, which was described in detail in our papers [20–24]. Generally, 30 μg proteins from Leu-d_3_-labeling cells were respectively mixed with 30 μg HGT or PBT proteins to separate on 12% SDS-PAGE. Gels were excised to in-gel digest and extract peptides, peptides were identified by LC-nanospray-tandem MS (MS/MS) on a QSTAR XL mass spectrometer (Applied Biosystems, USA). The parameters for database searching were mainly followed as our previous approaches [22, 23, 25]. The relative tissue protein expression level (SILAC ratio) was quantified by tracking pairs of labeling and unlabeling peptides from MS spectra. The differential expression protein was defined with its change ratio above 2 or below 0.5 times as a significantly up-regulated or down-regulated one between HGTs and PBTs, which was performed following bioinformatics analysis [23, 24].

### SAE1-specific siRNA, expression plasmid and cell transfection

SAE1-specific small interfering RNA (SAE1 siRNA) was designed to suppress SAE1 expression in human glioma cells. Three pieces of SAE1 siRNA sequences were designed to screen the most efficient one. SAE1 siRNA 1: 5′-AGA CAA CGA TGG TCA AAA A-3′, SAE1 siRNA 2: 5′-GTG CTT CTT GTC GGC TTG A-3′, SAE1 siRNA 3: 5′-AGC GAG CTC AGA ATC TCA A-3′. The non-target oligonucleotides (NC) (5′-UUC UCC GAA CGU GUC ACG U-3′) were taken as control. All RNA oligomers were synthesized by the Guangzhou RiboBio Company (China). 100 nM SAE1 siRNA was transiently transfected for one well of a 6-well plate for 48 h [4]. And the cholesterol-conjugated SAE1-specific siRNA for in vivo injection with mouse xenograft models was also ordered from RiboBio Company (China). The cholesterol-conjugated SAE1 siRNA was injected with mouse xenograft models [26]. which was also ordered from RiboBio Company (China).

The SAE1 cDNA (gi 225543279) was cloned into an expression vector pEZ-M13, which included a Flag tag. The recombinant expression plasmid pFlag-SAE1 was verified to be correct by DNA sequencing. For each well of a 6-well plate, 2.5 μg pFlag-SAE1 plasmids were respectively transfected into U87, U251 cells to observe cell behavior [5], including cell growth, migration and cell cycle.

### Quantitative RT-PCR

The RT-PCR primers of SAE1 were designed to detect its gene expression level. SAE1: forward primers 5′-TGGAGCAGTGAGAAAGCAAAG-3′ and reverse primers 5′-GGAAGCAGGTCAGGACTAATAC-3′. The housekeeping gene GAPDH was taken as an internal reference. GAPDH forward primer: 5′-TGG AAG GAC TCA TGA CCA CA-3′ and reverse primer 5′-TTC AGC TCA GGG ATGACC TT-3′.

### Immunoprecipitation

Cells or tissues were collected to lyse with an immunoprecipitation (IP) buffer containing 20 mM Tris (pH 7.5), 150 mM NaCl, 1% Triton X-100, protease inhibitor cocktail and 20 mM N-ethylmaleimide. Then 2 mg cellular supernatant was incubated with the complex of anti-SUMO1 antibody (ab32058, Abcam) and protein-A beads (161–4013, Bio-Rad) overnight to enrich SUMOylated proteins [5]. As a background negative control, the primary antibody was replaced with the normal rabbit IgG (A7016, Beyotime, China) to eliminate the nonspecific protein binding with SUMO1. After washing 4 times with TBS buffer, protein complexes were eluted with sample-loading buffer (P0015, Beyotime) for Western blot detection.

### Western blot

Proteins were separated on a 10% or 12% SDS-PAGE gel to test the target protein level using specific antibodies. The specific primary antibodies included SAE1 (ab56957 & ab185552, Abcam), SUMO1 (ab32058, Abcam), Akt (4961, Cell signaling), p-Akt (4060, Cell signaling), CDK2 (ET1602–6, HuaBio), Cyclin D1 (2922, Cell signaling), p21 (ab109199, Abcam), Bcl-2 (ET1603–11, HuaBio) and active Caspase-3 (ET1602–47, HuaBio), which were used to detect SAE1-invoved cell signaling pathway. The corresponding secondary antibody was subsequently incubated for 1 h to visualize signals at room temperature. The mouse anti-β-actin antibody (TA-09, ZSGB) was taken for signal normalization.

### Cell proliferation

Cell proliferation was determined by cell counting kit-8 (CCK-8) assay. After cell transfection with SAE1 siRNA for 24 h, 2 × 10^3^ glioma cells were seeded into a 96-well plate to culture with 10% FBS-containing DMEM for 12-60 h. 10 μl of CCK-8 reagents was added to each well to incubate for 2 h at 37 °C [4], which was measured the optical density absorbance at wavelength of 450 nm.

#### Transwell assay

Cell migration under SAE1 siRNA treatment was detected by a transwell chamber apparatus (PIEP12R48, Millipore) as described before [4, 5, 21]. After being transfected with SAE1-specific siRNA for 48 h, 1 × 10^4^ mixed cells in 200 μl serum-free DMEM were seeded in the upper chamber of a transwell, and the bottom of the chamber was filled with 600 μl of DMEM containing 10% FBS. Cells on the upper side of the filter were removed after 24 h. The filter membrane was stained with crystal violet, and the number of the cells that remained adherent to the underside of the membrane were counted under an inverted microscope (Zeiss Axiovert). The quantity of viable non-migrating cells through the chamber was calculated based on the number that the initial alive inoculation cells subtracting the migrating cells. The number of viable non-migrating cells = (The initial inoculation cells - the initial inoculation cells × cell apoptosis ratio) - the migrating cells. Each assay was separately performed for three replicates and all experiments were repeated at least three times.

#### Wound healing assay

The glioma cells were seeded into 6-wells plate at an appropriate concentration. After being transfected with SAE1-specific siRNA or non-specific siRNA for 24 h, the cells were scratched with tips and washed with PBS three times to remove the detached cells. Cells were allowed to grown for 24 h and 48 h in Serum-free medium, during which time wound margins were photographed and migration was monitored using an inverted microscope [27]. Migration distances were calculated relative to the initial distance by Image J software.

### Cell apoptosis & cell cycle analysis

Cell apoptosis was detected using a double staining apoptosis detection kit with following the protocol (KGA106, Nanjing KeyGen Biotech Co., Ltd., China) [28]. Briefly, 5 × 10^5^ cells were seeded into each well in a 6-well plate. After cell transfection with SAE1 siRNA for 48 h, cells were collected to incubate with Annexin V-FITC and PI for 15 min to detect cell apoptosis by flow cytometry. Similarly, cell cycle was analyzed by flow cytometry under SAE1 knockdown in cells [28].

### Subcutaneous glioma xenograft

All animal experiments were approved and conducted by the Institutional Animal Care and Treatment Committee of Sichuan University, China. The exponentially growing glioma cells were harvested to wash with serum-free medium, and mixed in serum-free medium at a concentration of 1 × 10^7^/ml. Then 100 μl of cell suspension was injected into male BALB/c-nude mice (5 weeks old, each weighing 18-20 g) subcutaneously. All mice were handled in strict accordance with good animal practice [5, 21].

Totally 4 groups of glioma xenograft were performed to observe SAE1-induced tumorigenesis, and 5 nude mice were included in each group. U87 glioma cells with or without SAE1 overexpression were injected into the right flank region of mice. Since palpable tumors arose after inoculation of glioma cells about for 2 weeks, the tumor volume and body weight were measured every 3 days until total observation up to 8 times [5, 21]. Tumor volumes were averagely calculated from 5 tested mice. Tumor growth was measured every 3 days using calipers, and the tumor volume was calculated by the eq. V = 0.52 × length×width^2^.

Another two nude mice groups were both subcutaneously pre-inoculated with U87 cells to drive glioma xenograft formation until tumor volume approximate to 100 mm^2^, in which tumor size and was measured every 3 days in response to treatment in vivo for 5 times. The 5 nmol cholesterol-conjugated SAE1 siRNA or nontargeting siRNA was intra-tumoral injection in each tumor-bearing mouse of the two groups respectively once every 3 days and tumor volumes were determined by the following formula: volume = (length × width^2^) × 0.52 [29]. Finally all mice were killed to measure tumor weight, and detected related protein levels in tumor tissues.

### Statistical analysis

All data are expressed as means±S.D. Statistical analysis among groups using the two-tailed Student’s t-test in GraphPad Prism 6.0, *p* < 0.05 was regarded as significant difference.

## Results

### SAE1 upregulation correlates with poor survival of glioma patients

We identified SAE1 upregulation in glioma through the differential proteome dissection between HGTs and PBTs, in which totally 70 differential proteins with at least 2-times’ changes (Additional file 4: Table S1). The differential proteins included 23 upregulated and 47 downregulated ones in HGTs compared with PBTs. Then we applied database Gene ontology (GO, http://www.geneontology.org/), Kyoto Encyclopedia of Genes and Genomes (KEGG, http://www.kegg.jp/kegg/pathway.html) and DAVID (http://david.abcc.ncifcrf.gov/) to classify the changed proteins. These proteins are mainly involved in biological process, cellular component, molecular function and some modification process (Additional file 1: Figure S1). For instance, we noticed that SAE1 protein, mediating protein SUMOylation, was increased to 7.42-fold level in HGTs compared with PBTs from the SILAC-based LC/MS analysis (Additional file 4: Table S1).

**Fig. 1:**
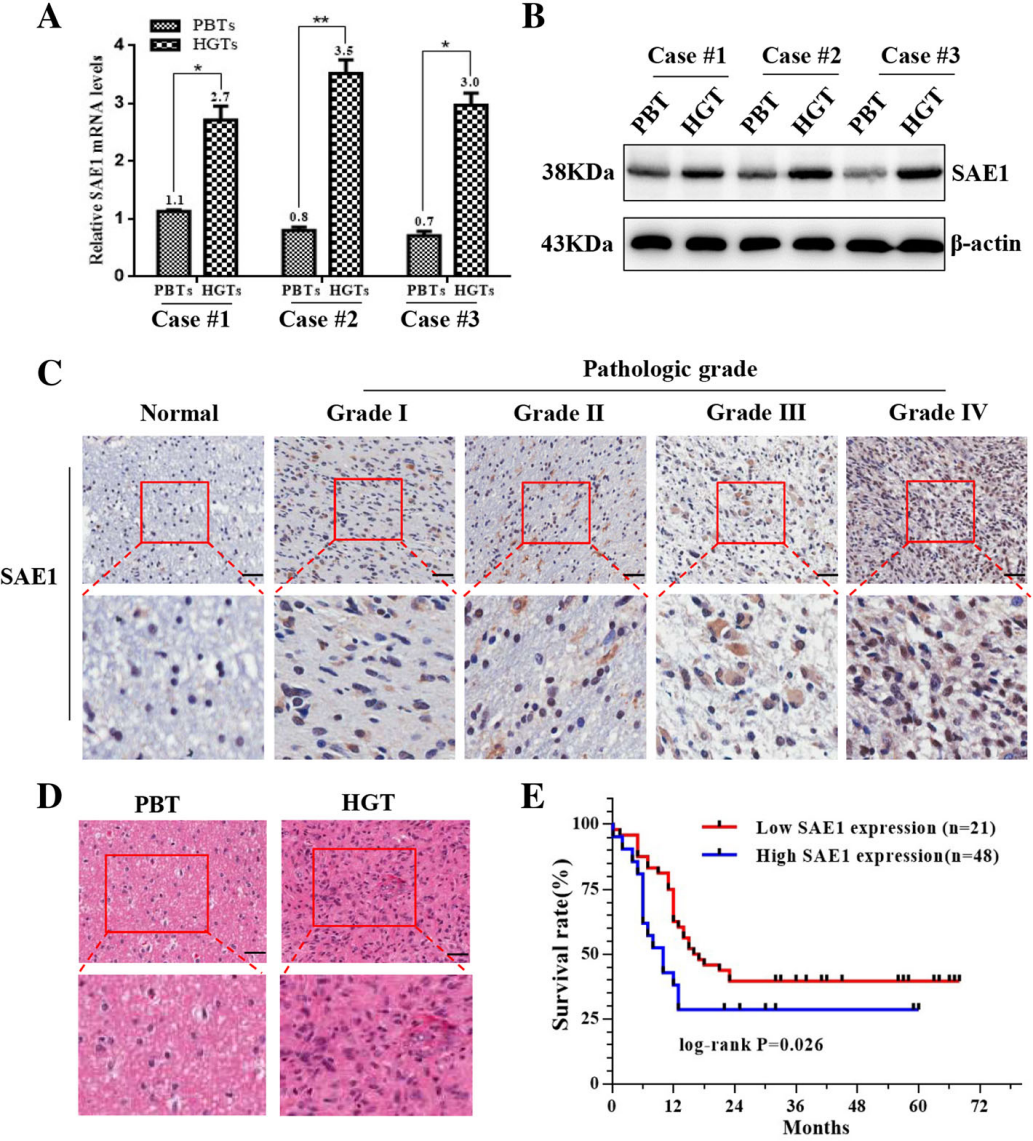
SAE1 upregulation in human glioma tissues correlates with the patient’s survival. **a** SAE1 mRNA expression was measured by qRT-PCR between 3 random chosen HGTs and their paired PBTs. The relative transcript levels were normalized to GAPDH. *p < 0.05, **p < 0.01. **b** SAE1 protein levels were shown by Western blot in 3 pairs of HGTs and PBTs. HGTs: human glioma tissues; PBTs: para-cancerous brain tissues. **c** Immunohistochemical staining of SAE1 in PBTs and giloma tissues with different pathologic grades (400×). **d** The hematoxylin-eosin (H&E) staining of glioma tissue (400×). **e** Kaplan-Meier overall survival curves for glioma patients based on SAE1 levels. Glioma patients with high expression of SAE1 had a worse postoperative overall survival

Subsequently SAE1 levels were monitored in three pairs of HGTs and PBTs by real-time PCR (Fig.1a) and Western blot detection (Fig.1b). It was consistent to confirm the up-regulated SAE1 at mRNA and protein levels in the glioma tissues.

To validate the expression level of SAE1 on a certain number of glioma tissues, we further examined SAE1 protein by IHC between 69 HGTs and 12 PBTs. The SAE1 protein showed a much higher expression in 69 gliomas with averagely scoring 6.49 compared with 12 counterparts (*p* < 0.05) (Table 1). In particular, SAE1 was high expression in 69.6% (48/69) human glioma tissues, with an average staining score 7.76 ± 0.32 and other 21 cases (30.4%, 10/69) showed low SAE1 expression with mean staining scores 3.19 ± 0.22. While except to one case, a marked low expression of SAE1 usually was tested in 91.7% (11/12) PBTs with an average staining score 1.46 ± 0.31. Moreover, SAE1 level is stronger in the higher pathologic grade of gliomas. The typical IHC images of SAE1 expression with different pathologic grade were displayed in Fig.1c. We also paid attention to the association between SAE1 expression and the clinicopathologic features of human gliomas. The tumor tissues were collected from 69 glioma patients, including 37 males and 32 females with an age ranging from 32 to 73 years. Generally, more strong levels of SAE1 were detectable in the glioma tissues with higher pathologic grades (Table 2, Fig. 1c). A high-expression of SAE1 was significantly associated with the grade III-IV gliomas, whereas a low-expression of SAE1 was more often associated glioma with grade I-II (*P* = 0.032) (Table 2). The expression level of SAE1 has no relationship with patient’s gender and age. Meanwhile, we analyzed SAE1 expression and the survival time of glioma patients. Kaplan-Meier analysis demonstrated a significantly longer overall survival was observed for patients with low level of SAE1, and a shorter overall survival was in those with high level of SAE1 (*P* = 0.026) (Fig.1e). We also used two online databases to analyze the expression level of SAE1 protein in various cancer tissues to verify our results on glioma. The Human Protein Atlas (HPA) database, which is dedicated to provide tissue and cell distribution information for all human proteins (https: //www.proteinatlas.org/). The UALCAN database, which is interactive web-portal to perform in-depth analyses of TCGA gene expression data (http://ualcan.path.uab.edu). The bioinformatics analysis of SAE1 from these two databases demonstrated that high expression of SAE1 is associated with poor survival and prognosis of patients suffering from glioma, liver cancer, renal cancer and thyroid cancer (Additional file 2: Figure S2A, S2B). These overall survival results through online database analysis were consistent with clinical prognosis survey for glioma patients. As a conclusion, a high level of SAE1 expression indicates a high degree of glioma grade, and a short overall survival for glioma patients.

**Table 1:** SAE1 expression profiling between 69 HGTs and 12 PBTs

Immuno-reactivity	HGTs (*n* = 69)		PBTs (*n* = 12)		*p* value
	Percentage	Average score	Percentage	Average score	
Total	100% (69/69)	6.49 ± 0.34	100% (12/12)	1.73 ± 0.45	*p* < 0.001
Low (+)	30.4% (21/69)	3.19 ± 0.22	91.7% (11/12)	1.46 ± 0.31	
High (++)	69.6% (48/69)	7.76 ± 0.32	8.3% (1/12)	5	

*HGTs* human glioma tissues. *PBTs* para-cancerous brain tissues

The immunoreactivity differences between HGTs and PBTs groups were estimated using Student’s t-test

Percentage: (specific cases/total cases)

Low SAE1 level (+) was scored 1–4, while the high level (++) was more than 4 scores

**Table 2:** Correlations of SAE1 expression with glioma patient’s information

Clinical parameters	Patient number (n)	SAE1 expression		Average score	*p* value
		Low level (n)	High level (n)		
Total	69	21	48		
Gender					
Male	37	12	25	6.62 ± 0.51	0.685
Female	32	9	23	6.34 ± 0.43	
Age					
< 56	39	10	29	6.79 ± 0.44	0.135
≥ 56	30	11	19	7.43 ± 0.48	
Pathologic grade					
I-II	20	11	9	5.14 ± 0.75	0.032
III-IV	49	10	39	7.00 ± 0.40	

*p* value was calculated using Pearson χ2 test

Low expression: SAE1 staining was scored 1–4. High expression: SAE1 staining was scored more than 4

Pathologic grade: The pathologic grade based on World Health Organization (WHO) classification

### SAE1 knockdown decreases glioma cell proliferation and migration

**Fig. 2:**
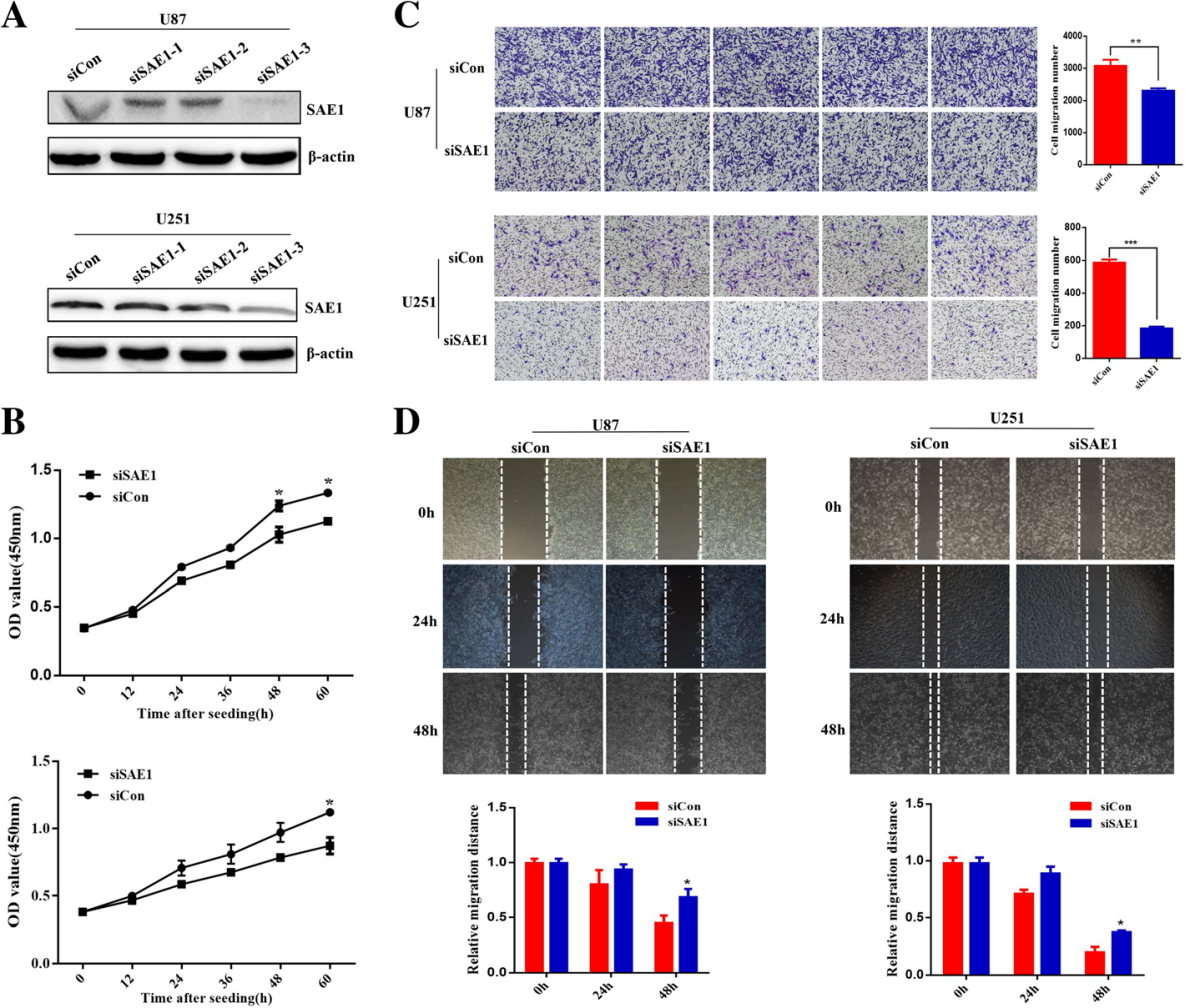
SAE1 knockdown decreases glioma cell proliferation and migration. **a** The interference effects of three specific SAE1 siRNAs in U87 and U251 cells. The siRNA-3 against SAE1 had the most effective gene inhibition. **b** SAE1 siRNA (siSAE1–3) decreases U87 and U251 cells proliferation. Cell proliferation was detected at transfection for 0, 12, 24, 36, 48, 60 h in glioma cells. Data are represented as the mean ± SD of three separate experiments. *p < 0.05. **c** The transwell assay was used to detect cell migration ability. Cells were observed at 24 h after transfection with 100 nM siSAE1–3 in U87 and U251 cells. **d** Cell migration was measured with wound healing assay after transfection for 24, 48 h. And cell migration distances were calculated relative to the initial distance before migration. siCon: non-targeting control siRNA. siSAE1: The SAE1-specific siRNA-3 (siSAE1–3). Data were presented as mean ± SD of three separate experiments. *p < 0.05, **p < 0.01

In order to explore SAE1 roles in glioma cell behavior, lose-of-function of SAE1 was respectively performed in U87 and U251 cells. We screened SAE1 siRNA sequence 3 (siSAE1–3) with most efficient gene interference in U87 and U251 cells by Western blot detection (Fig. 2a).

We further used siSAE1–3 to block cell endogenous SAE1 level to observe cell growth and migration. As expected, SAE1 knockdown significantly decreased cell proliferation by 19, 29% in U87 and U251 cells by 100 nM siSAE1–3 treatment for 60 h (*p* < 0.05) (Fig. 2b). Meanwhile, cell migratory capacity was weakened after SAE1 knockdown, which were revealed by the transwell assay and wound healing analysis (Fig. 2c-d). In order to exclude the apoptotic cells in the migration assay, we detected cell apoptosis after SAE1 siRNA treatment for 24 h. However, cell apoptosis was almost no absence (Additional file 3: Figure S3). So, cell migration number in SAE1-knockdown U87 and U251 cells was correspondingly decreased to 1.3 and 3.2-times compared with the control groups (*p* < 0.01). The ratio of viable non-migrating U87, U251 cells was 63, 89% in response with SAE1 siRNA treatment respectively (Fig. 2c). Moreover, the migration distance in SAE1-knockdown U87 and U251 cells was correspondingly decreased to 1.5 and 1.9 times compared with the control groups after SAE1 siRNA treatment for 24 h (*p* < 0.05) (Fig. 2d). Therefore, SAE1 knockdown in U87 and U251 cells both exhibited a significant suppression of cell proliferation and cell migration.

### SAE1 enhances Akt SUMOlyation and phosphorylation

As one subunit of the SUMO-activating enzyme SUMO E1, SAE1 is indispensable for protein SUMOylation [6]. Our recent study confirms the phosphorylated Akt is involved in glioma progression [4]. Previous studies have demonstrated Akt can be SUMOylated and Akt SUMOylation activates its kinase activity in cancer cells [30, 31]. In this study, we further examined whether the expression of SUMOylated AKT and phosphorylated Akt is related with SAE1 level between HGTs and the counterparts PBTs.

**Fig. 3:**
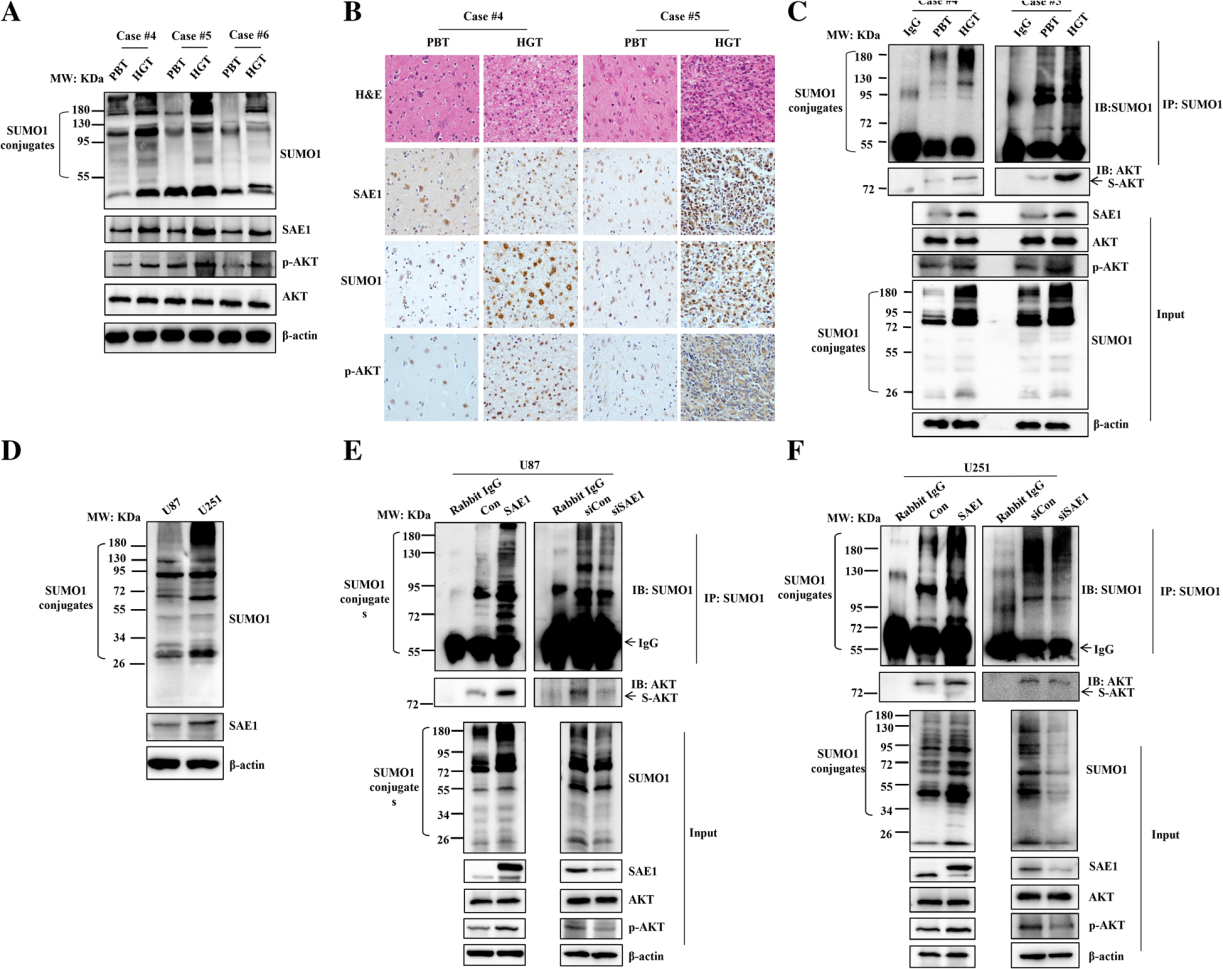
SAE1 protein enhances Akt SUMOlyation and phosphorylation. **a** The expression of SAE1, SUMO1, p-AKT in 3 random chosen HGTs and their paired PBTs. **b** The H&E staining and the immunohistochemical staining of SAE1, SUMO1, p-AKT in 2 cases of HGTs with a high pathologic grade and their paired PBTs. **c** The expression of SAE1, SUMOylated Akt, p-Akt (Ser473) and global SUMOylation were measured in 2 cases of HGTs with a high pathologic grade and their paired PBTs. HGTs: human glioma tissues; PBTs: para-cancerous brain tissues; case #4–6: glioma tissues respectively with the pathologic grade IV, III and I. **d** The levels of SAE1 and global SUMO1 modification were detected in U87 and U251 cells. (**e**-**f**) SAE1 activates Akt SUMOylation and phosphorylation (Ser473) in glioma cells. After the plasmid SAE1 or siSAE1 were transfected into U87 or U251 cells for 48 h, cell pellets were collected to extract cellular lysates to enrich SUMOylated proteins by IP, from which the protein were eluted to detect SUMO1 modified proteins and SUMOylated AKT. Cell lysates were also detected by Western blotting respectively with anti-SUMO1, anti-SAE1, anti-AKT, anti-p-AKT and β-actin antibodies. S-AKT: the SUMOylated AKT protein. p-AKT: phosphorylated Akt. Con: pFlag empty vector as a mock control. SAE1: pFlag-SAE1. siCon: non-targeting control siRNA. siSAE1: The SAE1-specific siRNA3. IP: immunoprecipitation, IB: immunoblot, Input: same account of cell lysate to load

Based on the expression levels of SAE1, Phospho-AKT (p-AKT) and global SUMO-1 levels (SUMO1 conjugates) of the 3 different grades of glioma tissues, generally their levels are relatively lower in the lower pathologic grade I of glioma than the higher grade III and IV (Fig. 3a). So, we reselected high pathologic grades of two glioma paired tissues (case #4 and case #5) to perform the experiments by IHC and co-IP assay. The IHC staining intensity showed that SAE1, SUMO1 and p-AKT proteins were higher in HGTs (Fig.3b). And the expression distribution of SAE1, SUMO1 and p-AKT in glioma tissues were mainly located in nuclear and cytoplasm (Fig.3b). At the same time, the protein extract from glioma tissues were immunoprecipitated with anti-SUMO1 antibody and immunoblotted with anti-Akt antibody. Except to SAE1 upregulation, the SUMOylated Akt and phosphorylated Akt were both increased in HGTs (Fig.3c). Meanwhile, the global protein SUMOylation was enhanced in HGTs compared to PBTs (Fig. 3c). These confirmed our conclusion that the expression of SAE1 is positively correlated with Phospho-AKT (p-AKT) and global SUMO-1 levels in glioma tissues.

Next we analyzed associations of SAE1 expression, Akt SUMOylation and phosphorylation in glioma cells. We chose U87 and U251 to perform the experiment, the endogenous SAE1 and global SUMO-1 modification in U87 cells were relatively lower compared with U251 cells (Fig.3d). The results of co-IP and Western blot assay showed that a high expression level of SAE1 enhanced the Akt SUMOylation and Akt phosphorylation (Ser473) in U87 cells (Fig. 3e). While SAE1 knockdown by SAE1-specific siRNA decreased Akt SUMOylation and Akt phosphorylation (Ser473) in U87 cells (Fig.3e). This was also confirmed in U251 cells (Fig. 3f). Therefore, SAE1 activates Akt SUMOylation and Akt phosphorylation (Ser473) in glioma.

Therefore, we found the expression of SUMOylated Akt and p-Akt are positively correlated with SAE1 level in glioma tissues and cells.

### Suppression of SAE1 induces cell cycle arrest and cell apoptosis

**Fig. 4:**
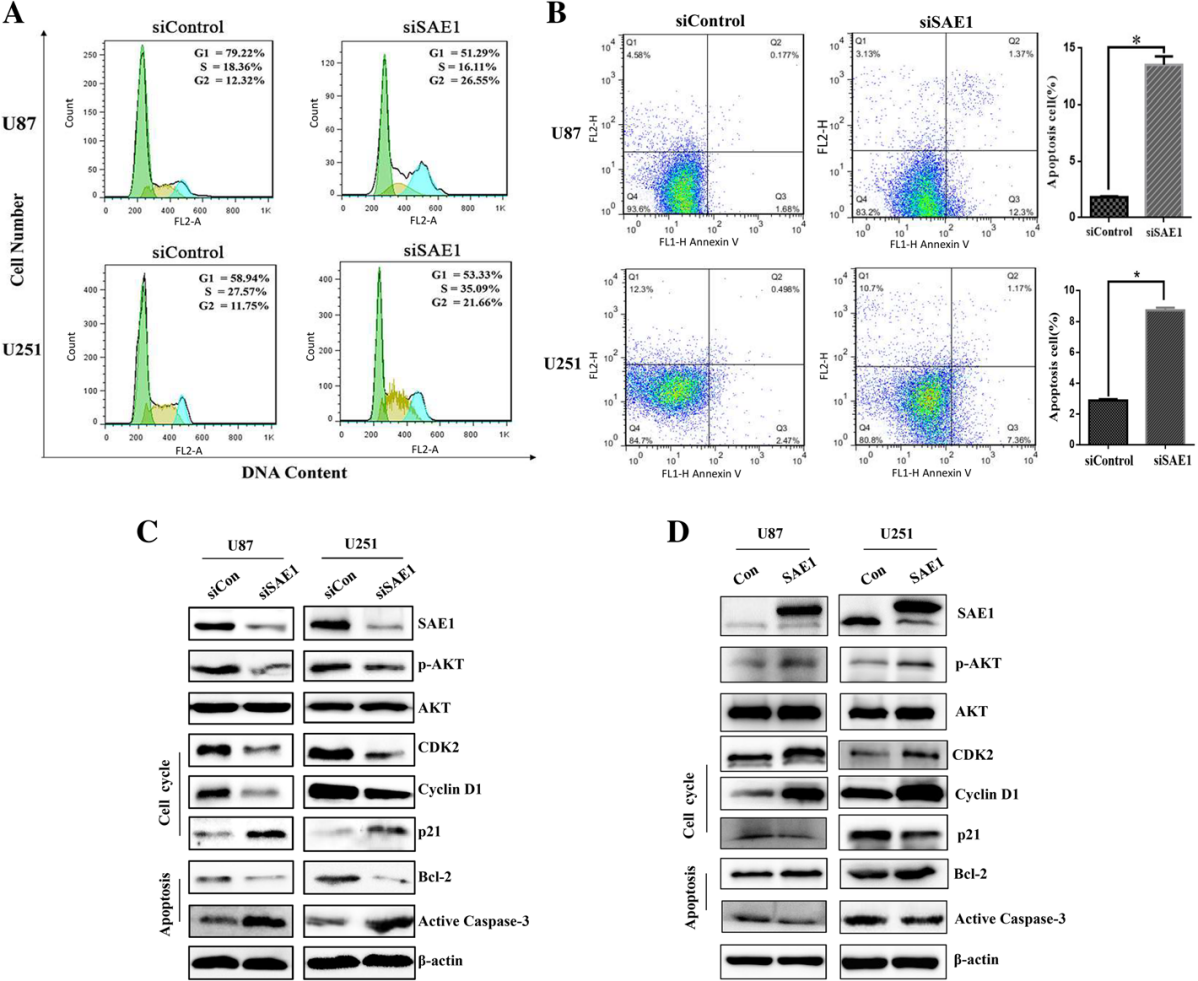
SAE1 knockdown induced G2 phase arrest and apoptosis of glioma cells. **a** SAE1 knockdown induced G2 phase arrest of glioma cells. The specific SAE1 siRNA3 was transfected into U87 and U251 cells for 48 h, following cells were staining with propidium idodide (PI) and assayed by flow cytometry. Quantification of cell cycle distribution was shown in the right panel. Data were presented as mean ± SD of three separate experiments. **b** SAE1 silencing induced apoptosis of U87 and U251 cells. Cell apoptosis was detected by Annexin V-PI double staining and flow cytometry analysis. The proportion of early apoptotic cells (annexin V positive) and late apoptotic cells (Annexin V and PI positive cells) were shown. Data were presented as mean ± SD of three separate experiments. *p < 0.05. **c** The expression of SAE1, AKT signaling pathway proteins, typical cell cycle-related proteins (CDK2, Cyclin D1, p21) and typical apoptosis-related proteins (Bcl-2, active Caspase-3) were detected by Western blot after siSAE1 treatment for 48 h. Each has the expression of β-actin as internal control. siCon: non-targeting siRNA. siSAE1: The SAE1 siRNA3 that specifically inhibits SAE1. **d** The AKT signaling pathway proteins and several typical cell cycle or apoptosis-related proteins were changed due to SAE1 overexpression. The β-actin was taken as internal control. Con: pFlag empty vector as a mock control. SAE1: pFlag-SAE1 transfection

Based on SAE1 downregulation responsible for cell growth inhibition (Fig. 2c), we further investigated SAE1-mediated glioma cell behaviors and molecular mechanism, including cell cycle distribution and cell cycle relative protein changes. We measured SAE1 knockdown-induced cell cycle and cell apoptosis by flow cytometry assay (Fig.4a-b). After SAE1 knockdown by siSAE1–3 treatment for 48 h in U87 and U251 cells, cell cycle arrest appeared an accumulation of G2 phase (Fig. 4a). And cell apoptosis was apparently up to 11.7 and 5.8% (Fig. 4b).

At the same time, AKT signaling pathway proteins, several cell cycle-related proteins (CDK2, Cyclin D1, p21) and apoptosis-related proteins (Bcl-2, active Caspase-3) were changeable in response upon SAE1 suppression. The p-Akt, CDK2, Cyclin D1 and Bcl-2 were significantly decreased, while p21 and active Caspase-3 were increased (Fig. 4c). Therefore SAE1 silencing inhibits glioma cell proliferation and induces cell apoptosis by regulating Akt-involved cell cycle protein level and cell cycle distribution. Meanwhile, SAE1 overexpression enhanced p-Akt, CDK2, Cyclin D1 and Bcl-2 levels in U87 and U251 cells (Fig.4d).

### SAE1 promotes glioma development in vivo

**Fig. 5:**
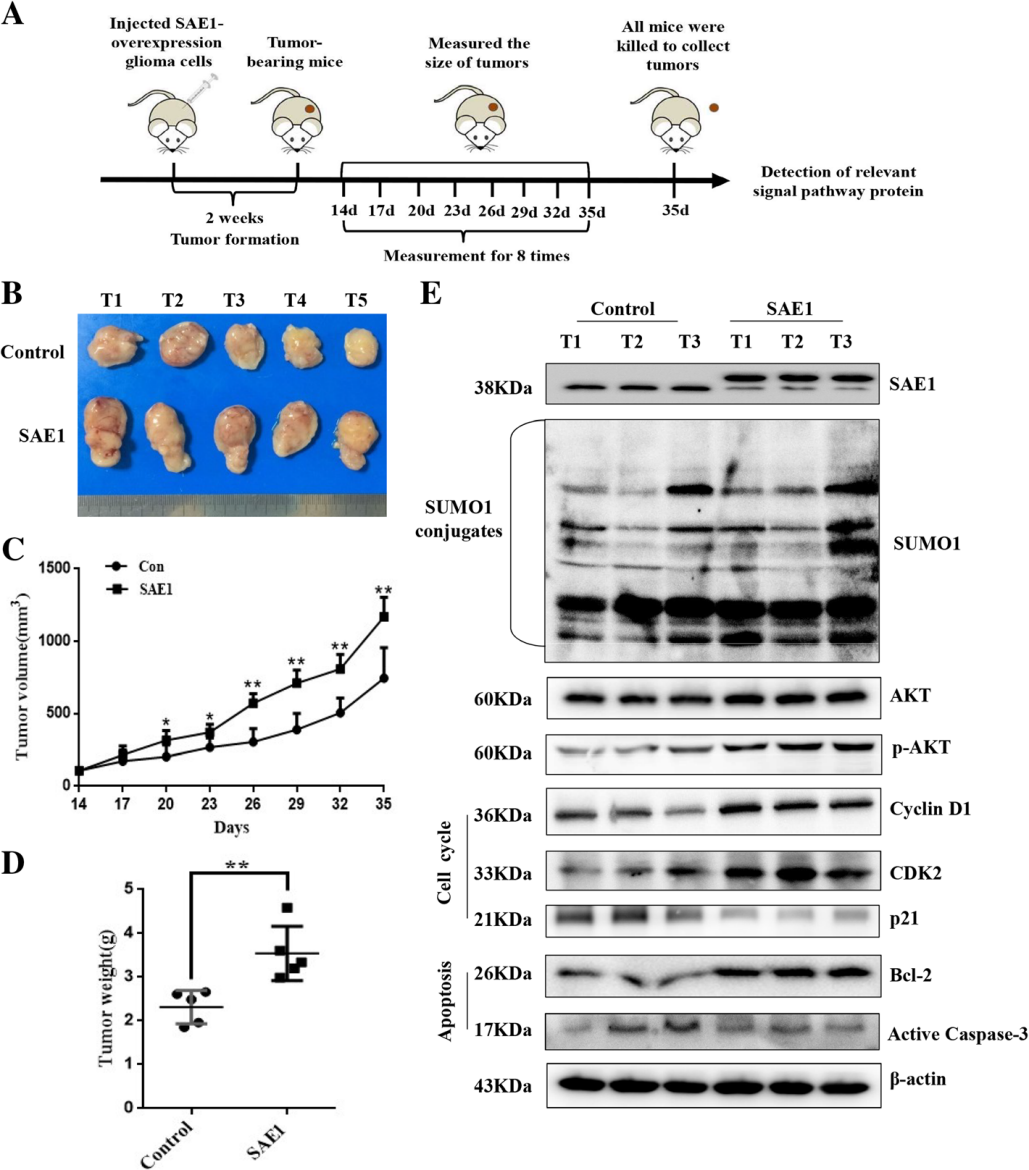
SAE1 promotes glioma development in vivo. **a** A schematic diagram was recorded the dates of treatments in SAE1-overexpressing mice. **b** The tumors isolated from xenograft nude mice, which were injected with pFlag-SAE1-overexpressing U87 cells, were much bigger than the control group injected with pFlag-containing U87 cells. After the tumor growth for 21 days (cell injection for 35 days), nude mice were killed to isolate tumor. **c** Tumor growth curves of xenograft nude mice. Data were presented as mean ± SD, *p < 0.05, **p < 0.01. **d** The average weight of the dissected tumors (on the 35th day) was much heavier than the control group. Data were presented as mean ± SD, *p < 0.05, **p < 0.01. **e** The expression of SAE1, AKT signaling pathway proteins, typical cell cycle-related proteins (CDK2, Cyclin D1, p21) and typical apoptosis-related proteins (Bcl-2, active Caspase-3) in random chosen nude mouse xenografted tumors. SAE1: nude mice inoculated with pFlag-SAE1-overexpressing U87 cells. Con: the control group, nude mice inoculated with pFlag-containing U87 cells. S-AKT: the SUMOylated AKT protein

SAE1-induced glioma development and molecular events were further confirmed in glioma xenograft mice. About 14 days after injection of SAE1-overexpressing glioma cells in nude mice, palpable tumors were observed approximately with 103 mm^3^, and subsequently the tumor size was totally monitored for 7 times from then on (Fig. 5a). After 20 days of injection, the volume of tumors was gradually enlarged. The mean tumor volume of glioma xenograft was 347 ± 31.5 mm^3^ in SAE1-overexpressing group after cell inoculation for 20 days, which was larger than the control group volume with 273 ± 36.4 mm^3^. At the 35 days’ cell injection time, the average tumor size of SAE1-overexpressing mice was up to 1170.8 ± 135.2 mm^3^, which was 1.44-fold large as the control with 814.3 ± 196.7 mm^3^ (*P* < 0.01, Fig. 5b-c). Finally, all mice were killed to measure tumor weight and detected related protein levels in tumor tissues at the 35 days’ cell injection. The mean tumor weight from SAE1-overexpressing mice was 3.54 ± 0.62 g after cell injection for 35 days, which was almost 1.53-fold heavy as the control group with 2.31 ± 0.38 g (*P* < 0.01, Fig. 5d). The expression levels of global SUMOylation, SAE1, AKT, p-Akt, Cyclin D1, CDK2, p21, Bcl-2 and active Caspase-3 were increased in the SAE1-overexpressing glioma tissues (Fig. 5e). These results showed that SAE1 significantly promoted glioma development in mouse glioma xenograft models.

**Fig. 6:**
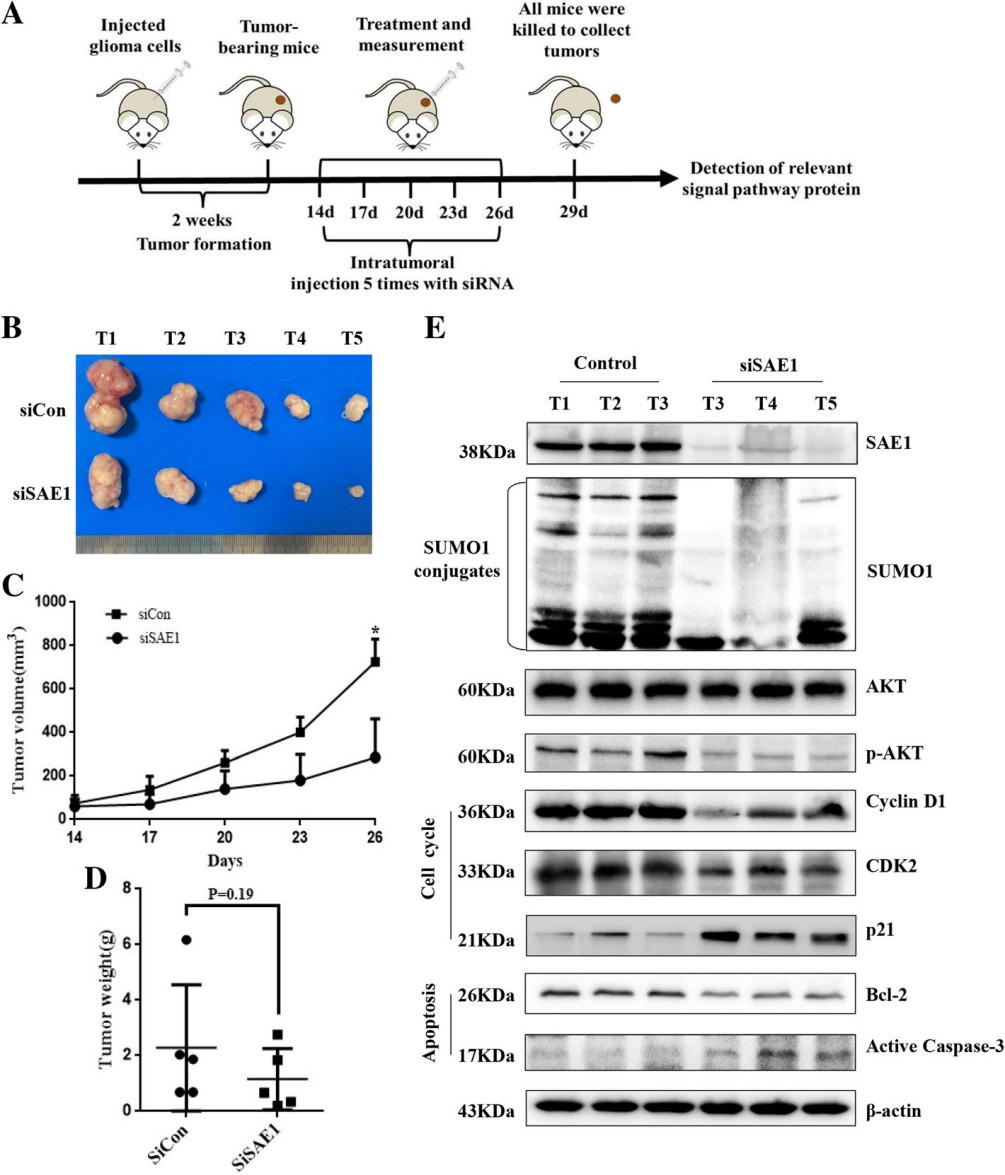
Silencing of SAE1 expression inhibits glioma development in vivo. **a** A schematic diagram was recorded the dates of treatments in mice. **b** The tumors isolated from xenografted nude mice, which were treated with siSAE1, were much smaller than the control group treated with siCon. After the tumor growth for 12 days (cell injection for 26 days), nude mice were killed to isolate tumor. **c** Tumor growth curves of xenograft nude mice. Data were presented as mean ± SD, *p < 0.05 is comparing with the control group. **d** The average weight of the dissected tumors (on the 29th day) was much smaller than the control group. Data were presented as mean ± SD. **e** The expression of SAE1, AKT signaling pathway proteins, typical cell cycle-related proteins (CDK2, Cyclin D1, p21) and typical apoptosis-related proteins (Bcl-2, active Caspase-3) in xenografted nude mice tumors. siSAE1: nude mice inoculated with U87 cells which were treated with SAE1 siRNA by intratumoral injection. siCon: nude mice inoculated with U87 cells which were treated with non-targeting siRNA by intratumoral injection. S-AKT: the SUMOylated AKT

To further verify the tumor suppression effect of SAE1 knockdown in vivo, we compared if the mouse tumor, which was inoculated U87 cells to form glioma xenograft, was suppressed by SAE1 siRNA treatment (Fig. 6a). After total 5 times treatment, the mean tumor volume for siSAE1 injection was almost decreased to 0.82-fold size of the siRNA control group (Fig. 6b-c). The mean tumor weight from mice with low SAE1 expression was 2.29 ± 2.40 g after cell injection for 29 days, which was almost 1.97-fold heavy as the control group with 1.16 ± 1.10 g (*P* = 0.19, Fig.6d). Similarly, the SAE1 downregulation-mediated signaling molecules were detected in tumor tissues by Western blot (Fig. 6e), among which their expressions were contrary to the SAE1 upregulation-induced protein changes (Fig. 5e). These results supported knockdown of SAE1 expression inhibits glioma development in vivo.

## Discussion

SUMOylation is a multistep enzymatic cascade reaction to bring SUMOs to Lys residue of substrate proteins. SUMOs, SAE1, SAE2, Ubc9 and some ligases perform their different function in the biochemical process of protein SUMOylation [6]. The three key enzymes are SUMO E1, Ubc9 and SUMO ligases, which are responsible for activation, transfer and ligation respectively in SUMOylation process. Emerging evidences have revealed that aberrant alterations of SUMOylation-associated enzymes may completely subvert proteins modulation involved in carcinogenic pathways, leading to abnormal cell proliferation, apoptosis resistance and metastatic potential [10]. For instance, the SUMO E1, a heterodimer of SAE1 and SAE2, catalyzes the first step of the SUMOylation cascade by promoting thioester-bond formation between the C-terminal glycine of SUMO and Cys173 of SAE2 in an ATP-dependent manner [12]. The higher levels of SUMO E1 are closely related with a poor survival of hepatocellular carcinoma patients [32]. Breast cancer patients with lower SAE1 and SAE2 expression have significantly lower instances of metastatic cancer and increased survival compared to patients with higher SAE1 and SAE2 levels [33]. In addition, knockdown of SAE1/2 makes synthetic lethality in tumors with high Myc expression or K-Ras mutations. Mechanistically, SAE inhibition switches a transcriptional subprogram of Myc from activation to repression [33, 34].

Glioma is induced by multiple factors including aberrant gene/protein abundance, abnormal protein modification and molecular signaling pathway, and hypernomic cell proliferation [35]. Attributed to resistance for glioma, the targeted agents against genetic hallmarks of glioma fail in clinical treatment, the median survival of glioma is less than 2 years [36]. Of particular interest are the recent reports that protein SUMOylation can regulate the development and progression of glioma [37, 38]. We focus on the SUMO-activating enzyme SAE1 roles on glioma and investigate whether targeting SUMOylation on mouse xenograft models can be as potential anti-cancer therapeutics?

In this study, we have discovered SAE1 upregulation is directly associated with glioma tumor stage and patient survival. The higher level of SAE1 means the worse survival for glioma patients. This is due to SAE1 upregulation promoting glioma cell growth and cell migration in vitro and in vivo through improving Akt SUMOlyation and Akt phosphorylation. While Akt SUMOlyation and phosphorylation are both decreased in response to SAE1 suppression on cell lines and glioma mouse models, in which cell cycle arrest and cell apoptosis are obviously changeable because of CDK2, Cyclin D1, Bcl-2 significantly decreased, and p21 and active Caspase-3 increased (Fig. 6). Therefore, SAE1 is a potential candidate marker for prognosis and biotherapy of glioma.

**Fig. 7:**
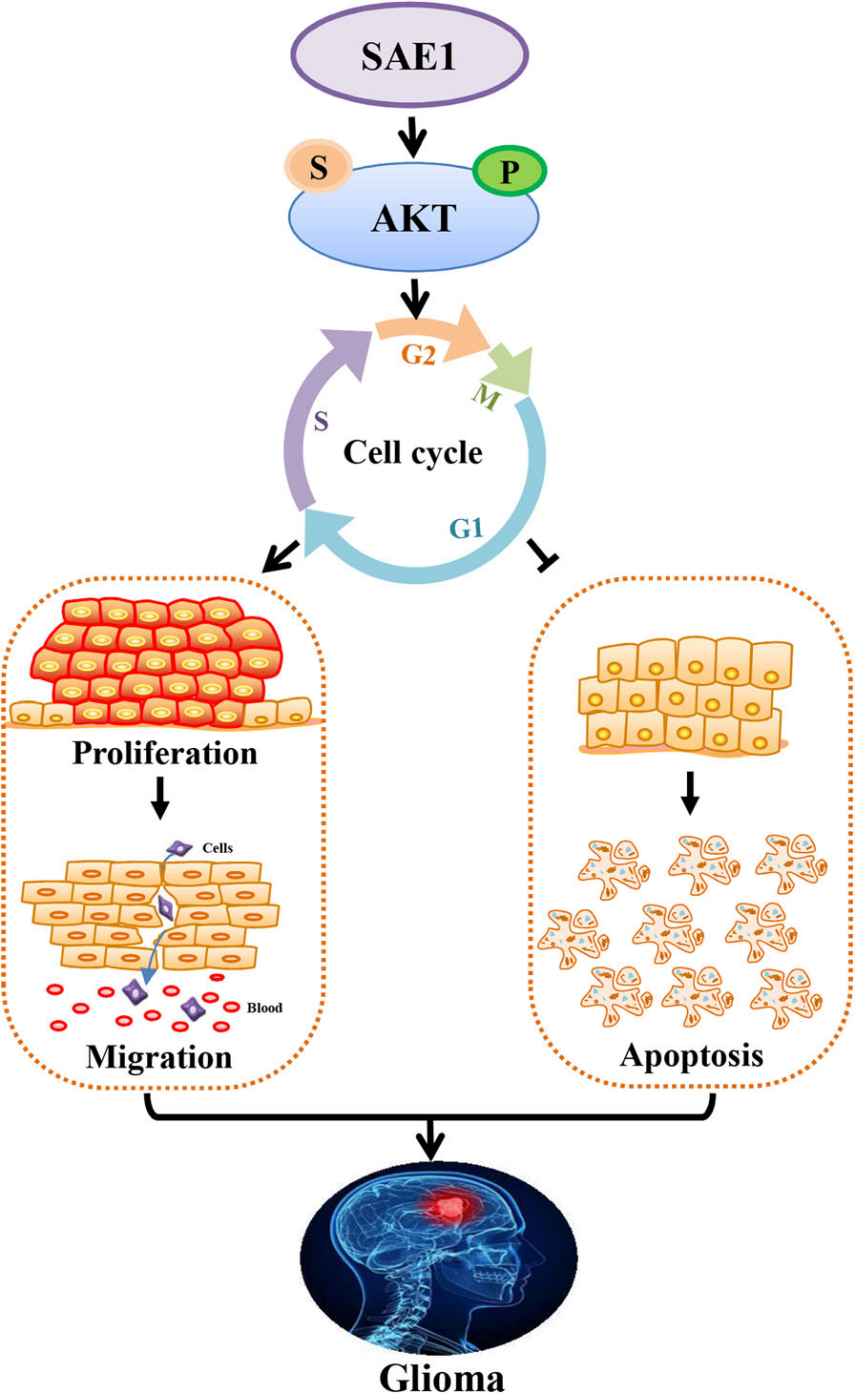
A schematic diagram illustrates the molecular mechanism of SAE1-induced glioma progression. SAE1 overexpression induces increase of the SUMOylation and Ser473 phosphorylation of AKT, which regulates cell cycle distribution and cell biological behaviors including cell proliferation, migration and cell apoptosis, these finally accelerate the occurrence and development of glioma.:SUMOylation;: Phosphorylation

Akt is a protein kinase involved in numerous essential biological processes, including cell proliferation, apoptosis, cell migration, metabolism, and tumorigenesis [39]. Akt-mediated signaling regulates cell growth in multiple cancers including glioblastoma [20, 26, 29, 30]. Furthermore, Akt has been reported to be modified by phosphorylation, ubiquitination, acetylation [39] and SUMOylation [30, 31]. Of course, the different forms of post translational modifications interplay. For instance, Akt SUMOylation is Akt phosphorylation dependent, and Akt SUMOylation increases Akt kinase activity [30, 31]. Our findings discover that SAE1 overexpression leads to increase of Akt SUMOylation and Akt phosphorylation (Ser473), which increases expression of CDK2, Cyclin D1, Bcl-2 and ultimately to promote glioma cell proliferation and progressin (Fig. 7).

Protein SUMOylation is a reverse process. We hypothesize the de-SUMOylation is prone to take place when SUMO activating enzyme SAE1 is suppressed, which finally inhibits to glioma cell growth in vitro and in vivo. In order to confirm this notion, we inhibited SAE1 expression by siRNA in U87, U251 cells to investigate cell cycle progression and cell proliferation. SAE1 knockdown induced cell cycle arrest via an accumulation of G2 phase and cell apoptosis, which were indicative through relative molecular events, including p-Akt, CDK2, Cyclin D1 and Bcl-2 decreased, while cell apoptosis percent was increased along with active Caspase-3 upregulated. Therefore, cell growth and cell migration were finally inhibited on glioma cell lines and glioma xenograft nude mice. These results contribute to understand the role of SAE1-regulated SUMOylation in cell cycle progression and glioma cell proliferation. Targeting SAE1 expression by small molecular inhibitors or RNAi is feasible way to discover novel drugs for glioma therapy.

Unlike ubiquitination, SUMOylation limits itself to one E1 activating enzyme (the heterodimer SAE1/2) and one E2 conjugase (Ubc9), thus targeting each of these components of the SUMO-conjugation machinery is likely to significant change in levels of SUMOylated proteins. So, SUMOylation pathway is an ideal drug target to overcome oncogenic mechanism [13, 40]. For example, ML-792 has been developed to selectively block SAE activity in treating MYC-amplified tumors [12]. Suppression of SAE2 by RNAi decreases cancer malignancy and enhances chemotherapy sensitivity in small cell lung cancer [41]. Aberrant protein SUMOylation relates with brain disease occurrence, such as brain ischemia [42] and Alzheimer’s disease [6], and it is also associated with ionization radiation resistance of glioma cells [43]. The enhanced SUMOylation of Smad4 is critical for DNA damage-induced activation of in-resistant glioma cells [43].

So far, targeting protein SUMOylation is still challenging for anti-cancer drug discovery due to it highly dynamic and rapid modification, which demands sensitive selection and excellent specificity for small molecular inhibitors.

## Conclusions

SAE1 upregulation enhances cell proliferation, migration by increasing the SUMOylation and phosphorylation of AKT to involve in relevant molecular signaling pathways, which finally accelerates the occurrence and development of glioma in vitro and in vivo.

## Additional files

Additional file 1: **Figure S1.** Bioinformatic analysis of 70 differential expression proteins in HGTs. (A) Gene ontology (GO) enrichment analysis of different expression proteins according to biological processes. (B) GO enrichment analysis of different expression proteins according to cellular component. (C) GO enrichment analysis of different expression proteins according to molecular function. (D) UP-Keyword analysis of different expression proteins. (TIF 1545 kb)

Additional file 2: **Figure S2.** SAE1 protein is correlated with the patient’s survival through integrated analysis of experimental data and online database. (A) Longer overall survival for lower grade glioma (LGG) patients with low SAE1 expression level from UALCAN database analysis. (B) Longer overall survival for different patients (e.g. Liver cancer, renal cancer and thyroid cancer) with low SAE1 expression level from HPA database analysis. (TIF 762 kb)

Additional file 3: **Figure S3.** SAE1 knockdown induced apoptosis of glioma cells. After being transfected with SAE1-specific siRNA for 24 h, cell apoptosis was detected by flow cytometry. siCon: non-targeting control siRNA. siSAE1: The SAE1-specific siRNA. ns: no significance. (TIF 1837 kb)

Additional file 4: **Table S1.** Differential expression proteins between human glioma tissues and para-cancerous counterparts (DOC 130 kb)

## Data Availability

All the dataset and materials generated and/or analyzed during the current study were available.

## Abbreviations

HGTs: Human glioma tissues
IHC: Immunohistochemistry
PBTs: Para-cancerous brain tissues
PTM: Post-translational modification
SAE1: the unique activating enzyme subunit1
siRNA: small interfering RNA
SUMO: the small ubiquitin-like modifier
Ubc9: Ubiquitin conjugating enzyme 9
