# Supplementary figures and images for: SAE1 promotes human glioma progression through activating AKT SUMOylation-mediated signaling pathways
Source: Cell Commun Signal. 2019 Jul 25;17:82. doi: 10.1186/s12964-019-0392-9 (PMC6659289; doi:10.1186/s12964-019-0392-9)

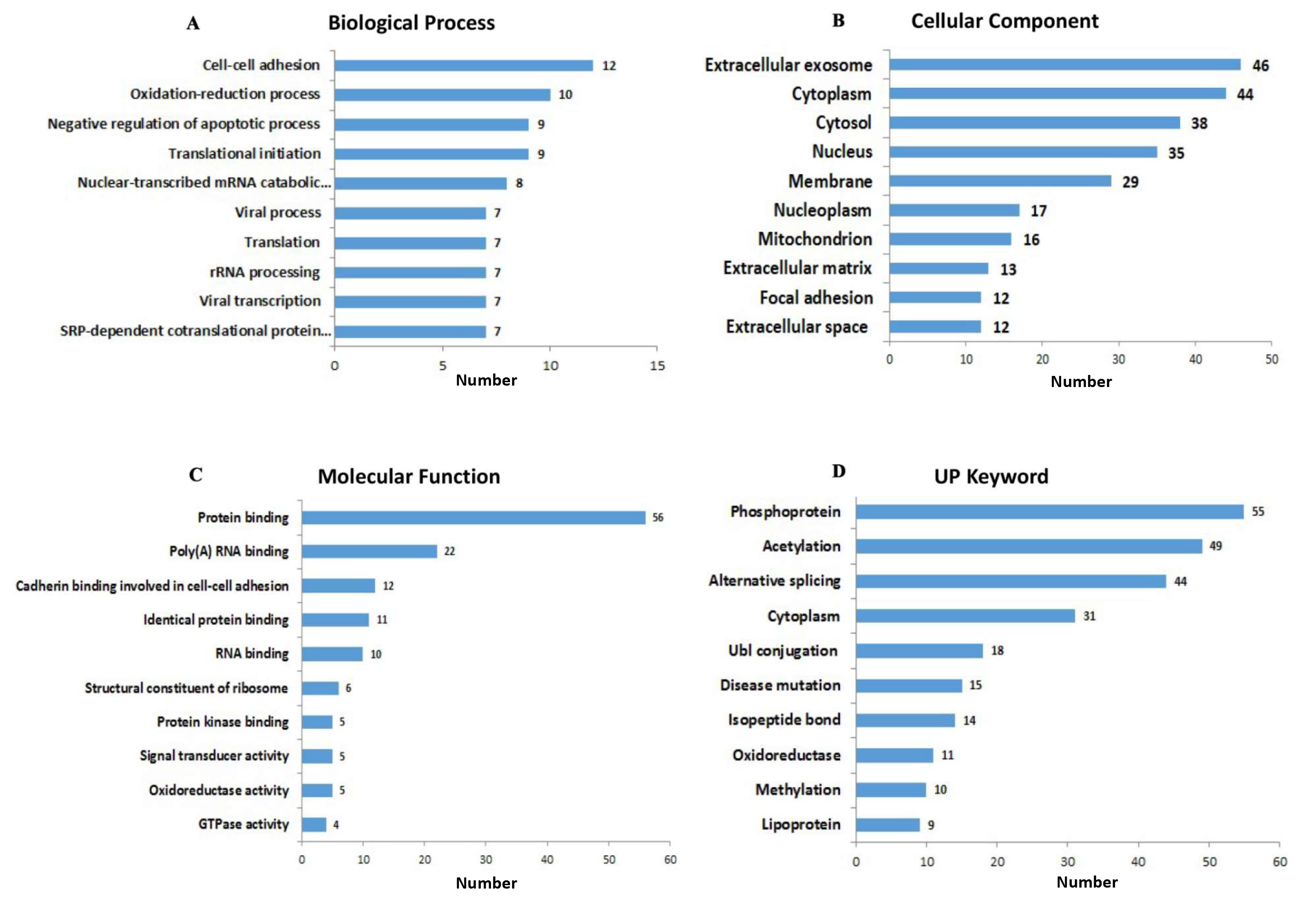

Supplement: Supplementary file 1 — Figure S1. Bioinformatic analysis of 70 differential expression proteins in HGTs. (A) Gene ontology (GO) enrichment analysis of different expression proteins according to biological processes. (B) GO enrichment analysis of different expression proteins according to cellular component. (C) GO enrichment analysis of different expression proteins according to molecular function. (D) UP-Keyword analysis of different expression proteins. (TIF 1545 kb) [file 12964_2019_392_MOESM1_ESM.tif]

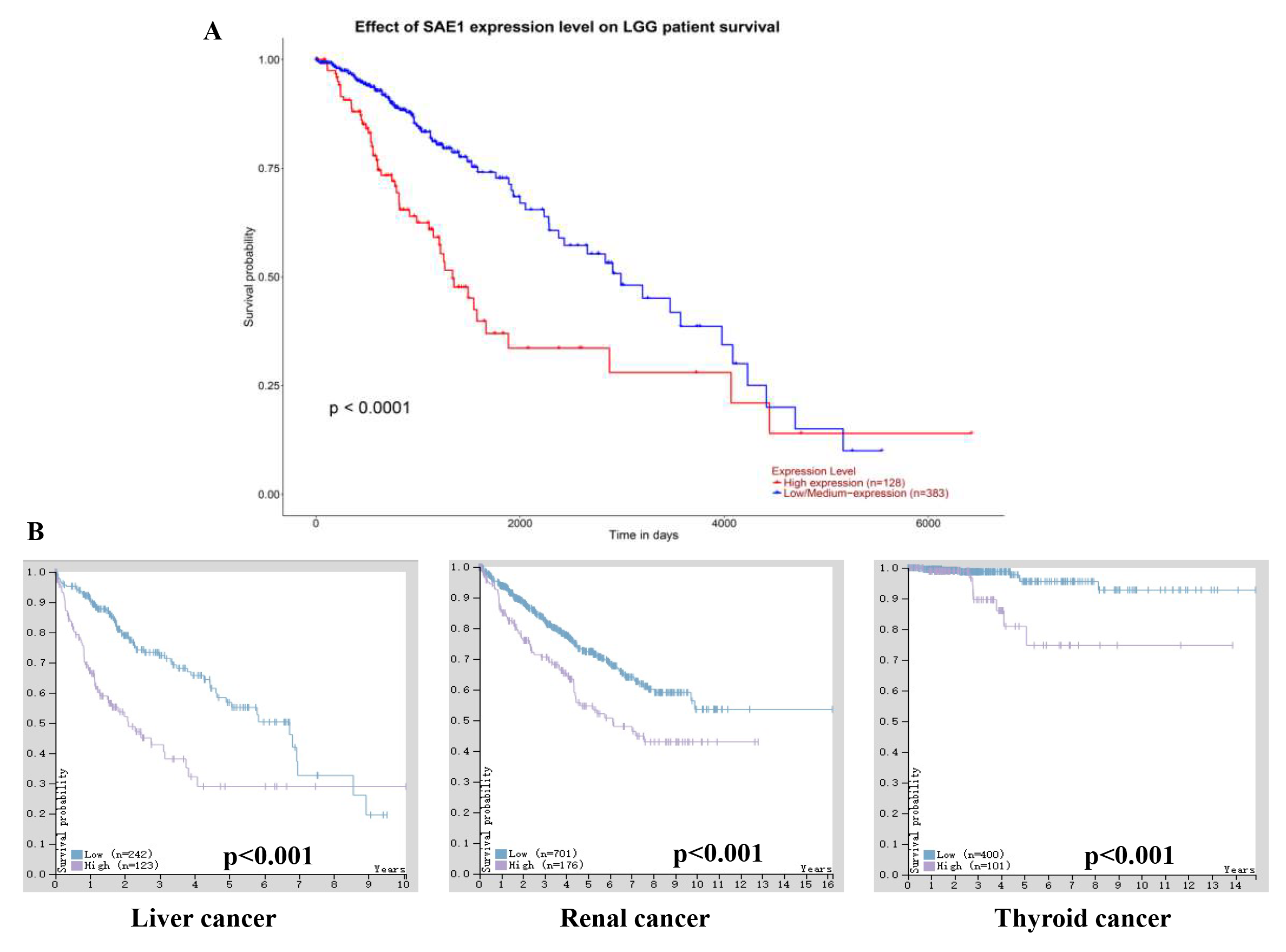

Supplement: Supplementary file 2 — Figure S2. SAE1 protein is correlated with the patient’s survival through integrated analysis of experimental data and online database. (A) Longer overall survival for lower grade glioma (LGG) patients with low SAE1 expression level from UALCAN database analysis. (B) Longer overall survival for different patients (e.g. Liver cancer, renal cancer and thyroid cancer) with low SAE1 expression level from HPA database analysis. (TIF 762 kb) [file 12964_2019_392_MOESM2_ESM.tif]

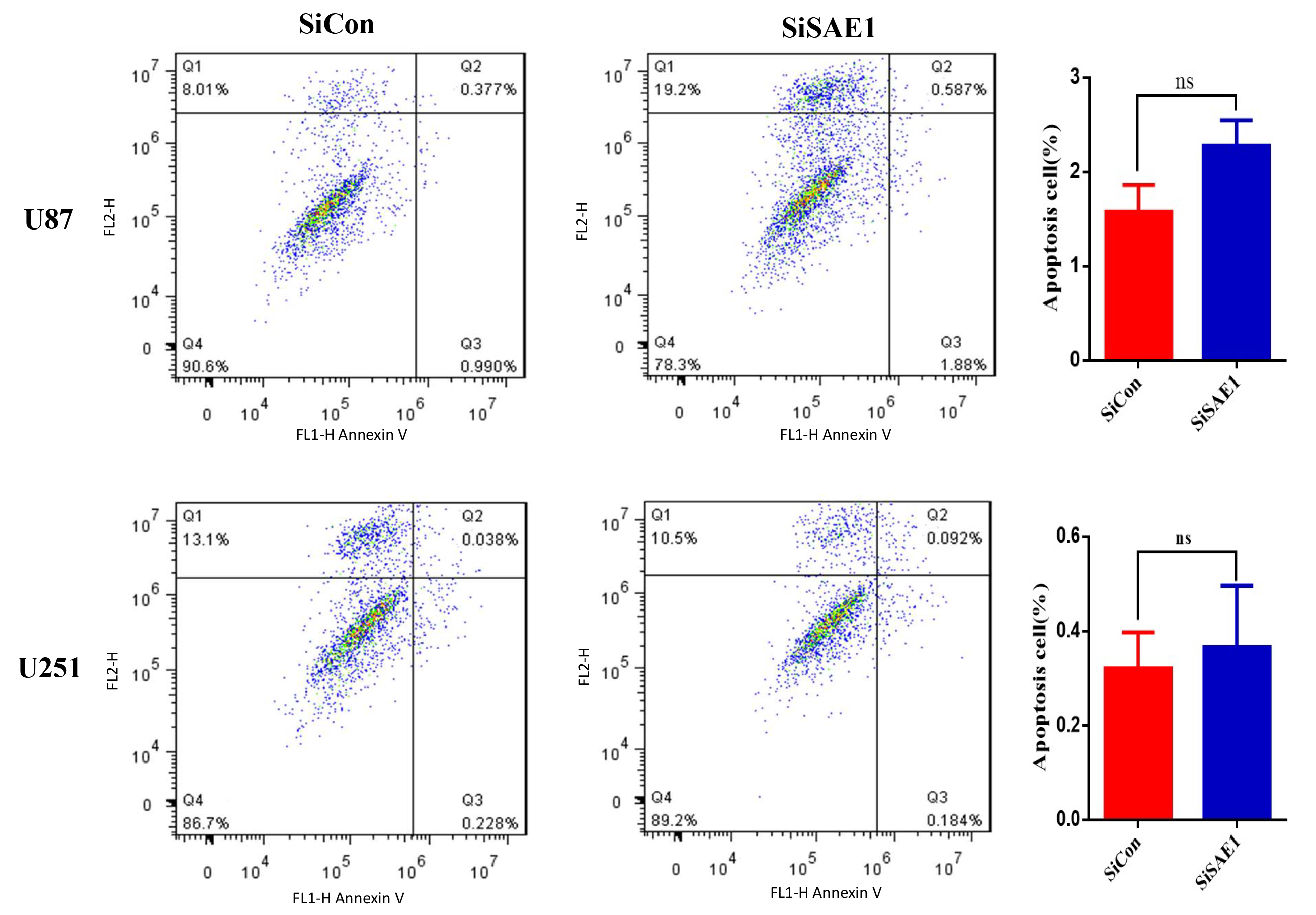

Supplement: Supplementary file 3 — Figure S3. SAE1 knockdown induced apoptosis of glioma cells. After being transfected with SAE1-specific siRNA for 24 h, cell apoptosis was detected by flow cytometry. siCon: non-targeting control siRNA. siSAE1: The SAE1-specific siRNA. ns: no significance. (TIF 1837 kb) [file 12964_2019_392_MOESM3_ESM.tif]
