# Supplementary material for: SAE1 promotes human glioma progression through activating AKT SUMOylation-mediated signaling pathways
Source: Cell Commun Signal. 2019 Jul 25;17:82. doi: 10.1186/s12964-019-0392-9 (PMC6659289; doi:10.1186/s12964-019-0392-9)
Supplement: Supplementary file 4 — Table S1. Differential expression proteins between human glioma tissues and para-cancerous counterparts (DOC 130 kb) [file 12964_2019_392_MOESM4_ESM.doc]

**Additional file 4: Table S1. Differential expression proteins between human glioma tissues and para-cancerous counterparts**

**Up-regulated proteins in glioma (23)**

| **Swiss-Prot No.** | **Gene name** | **Protein description** | **Theoretical Mr/pI** | **Function** | **Changes** |
| --- | --- | --- | --- | --- | --- |
| P09525 | ANXA4 | Annexin A4 | 35882.72/5.83 | Membrane fusion and exocytosis | 8.21 |
| Q9UBE0 | SAE1 | SUMO-activating enzyme subunit 1 | 38449.85/5.17 | Protein sumoylation | 7.42 |
| P62258 | YWHAE | 14-3-3 protein epsilon | 29173.90/4.63 | Phosphorylated protein HSF1 nuclear export to the cytoplasm | 3.39 |
| P05388 | RPLP0 | 60S acidic ribosomal protein P0 | 34273.51/5.70 | Equivalent of *E.coli* protein L10 | 3.02 |
| P60866 | RPS20 | 40S ribosomal protein S20 | 13372.71/9.95 | Ribonucleoprotein, ribosomal protein | 3.0 |
| Q07021 | C1QBP | Complement component 1 Q subcomponent-binding protein, mitochondrial | 31362.24 /4.74 | Bind to the globular "heads" of C1Q thus inhibiting C1 activation. | 2.59 |
| P26641 | EEF1G | Elongation factor 1-gamma | 31121.84/4.90 | Protein biosynthesis | 2.52 |
| P78417 | GSTO1 | Glutathione S-transferase omega-1 | 27565.86/6.24 | Participate in the biotransformation of inorganic arsenic | 2.52 |
| Q14694 | USP10 | Ubiquitin carboxyl-terminal hydrolase 10 | 87133.79/5.19 | Autophagy, DNA damage, DNA repair, Ubl conjugation pathway | 2.46 |
| Q9P0L0 | VAPA | Vesicle-associated membrane protein-associated protein A | 27893.23/8.80 | Bind to OSBPL3 and recruitment of VAPA to plasma membrane sites | 2.43 |
| P62244 | RPS15A | 40S ribosomal protein S15a | 14839.51/10.14 | Protein binding, structural constituent of ribosome | 2.33 |
| Q96CX2 | KCTD12 | BTB/POZ domain-containing protein KCTD12 | 35700.78/ 5.49 | Determine the pharmacology and kinetics of the receptor response | 2.32 |
| Q12792 | TWF1 | Twinfilin-1 | 40282.72/ 6.48 | Involve in motile and morphological processes | 2.27 |
| P11047 | LAMC1 | Laminin subunit gamma-1 | 177602.87/ 5.01 | Mediate attachment, migration and organization of cells into tissues during embryonic development | 2.26 |
| P22626 | HNRNPA2B1 | Heterogeneous nuclear ribonucleoproteins A2/B1 | 37429.70/8.97 | Involve in pre-mRNA processing, RNA nuclear export, subcellular location, mRNA translation and stability of mature mRNAs | 2.25 |
| P00387 | CYB5R3 | NADH-cytochrome b5 reductase 3 | 34234.86/7.18 | Desaturation and elongation of fatty acids, cholesterol biosynthesis, drug metabolism, and, in erythrocyte, methemoglobin reduction. | 2.15 |
| P50148 | GNAQ | Guanine nucleotide-binding protein G(q) subunit alpha | 42142.0/5.48 | Regulate B-cell selection and survival and is required to prevent B-cell-dependent autoimmunity | 2.13 |
| P61978 | HNRNPK | Heterogeneous nuclear ribonucleoprotein K | 50976.25/ 5.39 | Bind tenaciously to poly(C) sequences; involve in mRNA processing and mRNA splicing | 2.12 |
| P09543 | CNP | 2',3'-cyclic-nucleotide3'-phosphodiesterase | 45098.65 /8.73 | Participate in RNA metabolism in the myelinating cell | 2.1 |
| P09471 | GNAO1 | Guanine nucleotide-binding protein G(o) subunit alpha | 40050.5/5.34 | Involve as modulators or transducers in various transmembrane signaling systems | 2.1 |
| O60493 | SNX3 | Sorting nexin-3 | 18762.36/8.71 | Protein transport | 2.1 |
| Q00341 | HDLBP | Vigilin | 141455.65/6.43 | Cholesterol metabolic process; lipid transport | 2.06 |
| P60842 | EIF4A1 | Eukaryotic initiation factor 4A-I | 46153.93/5.32 | Protein biosynthesis | 2.05 |

**Down-regulated proteins in glioma (47)**

| **Swiss-Prot No.** | **Gene name** | **Protein description** | **Theoretical Mr/pI** | **Function** | **Changes** |
| --- | --- | --- | --- | --- | --- |
| P02794 | FTH1 | Ferritin heavy chain | 21225.64/5.31 | Iron ion transport for iron homeostasis | 2.02 |
| O15144 | ARPC2 | Actin-related protein 2/3 complex subunit 2 | 34333.02/6.84 | Regulation of actin polymerization and together with an activating NPF mediates the formation of branched actin networks | 2.04 |
| Q06830 | PRDX1 | Peroxiredoxin-1 | 22110.36/8.27 | Cell protection against oxidative stress by detoxifying peroxides and as sensor of hydrogen peroxide-mediated signaling events | 2.04 |
| P08670 | VIM | Vimentin | 53651.68/5.05 | Involve with LARP6 in the stabilization of type I collagen mRNAs for CO1A1 and CO1A2 | 2.05 |
| P04792 | HSPB1 | HSP beta-1 | 22782.52/5.98 | Play a role in stress resistance and actin organization | 2.08 |
| O60762 | DPM1 | Dolichol-phosphate mannosyltransferase | 29634.28/9.57 | Catalytic subunit of the dolichol-phosphate mannose synthase complex | 2.09 |
| P30048 | PRDX3 | Thioredoxin-dependent peroxide reductase | 27692.65/7.68 | Act synergistically with MAP3K13 to regulate the activation of NF-kappa-B in the cytosol | 2.1 |
| P62277 | RPS13 | 40S ribosomal protein S13 | 17222.31/10.53 | Structural constituent of ribosome | 2.11 |
| O75821 | EIF3G | Eukaryotic translation initiation factor 3 subunit G | 35611.02/5.87 | Protein biosynthesis | 2.13 |
| O15394 | NCAM2 | Neural cell adhesion molecule 2 | 93046.27/5.44 | Play roles in selective fasciculation and zone-to-zone projection of the primary olfactory axons | 2.13 |
| O14594 | NCAN | Neurocan core protein | 143093.23/5.22 | Modulate neuronal adhesion and neurite growth during development | 2.13 |
| P09382 | LGALS1 | Galectin-1 | 14715.70/5.30 | Regulate apoptosis, cell proliferation and cell differentiation | 2.18 |
| O43759 | SYNGR1 | Synaptogyrin-1 | 25455.61/4.50 | Regulate exocytosis | 2.19 |
| Q09666 | AHNAK | Neuroblast differentiation-associated protein AHNAK | 629101.22/5.80 | Required for neuronal cell differentiation | 2.24 |
| P24539 | ATP5F1 | ATP synthase subunit b, mitochondrial | 28908.03/9.37 | Mitochondrial membrane ATP synthase | 2.28 |
| P49321 | NASP | Nuclear autoantigenic sperm protein | 85237.72/4.26 | Cell cycle, DNA replication, Protein transport | 2.28 |
| P30041 | PRDX6 | Peroxiredoxin-6 | 25034.99/6.00 | Catalyze the reduction of hydrogen peroxide and organic hydroperoxides | 2.3 |
| P37840 | SNCA | Alpha-synuclein | 14460.16/4.67 | Regulate dopamine release and transport; reduce neuronal responsiveness to various apoptotic stimuli | 2.3 |
| P11233 | RALA | Ras-related protein Ral-A | 23566.79/ 6.66 | Gene expression, cell migration, cell proliferation, oncogenic transformation and membrane trafficking | 2.31 |
| Q01105 | SET | Protein SET | 33488.88/4.22 | Apoptosis, transcription, nucleosome assembly and histone chaperoning | 2.32 |
| P37837 | TALDO1 | Transaldolase | 37540.13/6.36 | Important for the balance of metabolites in the pentose-phosphate pathway | 2.33 |
| P63104 | YWHAZ | 14-3-3 protein zeta/delta | 27745.10/4.73 | Anti-apoptosis, signal transduction, mRNA metabolic process | 2.38 |
| Q9Y281 | CFL2 | Cofilin-2 | 18736.62/ 7.66 | Controls reversibly actin polymerization and depolymerization in a pH-sensitive manner. | 2.39 |
| P09874 | PARP1 | Poly [ADP-ribose] polymerase 1 | 113083.79/8.99 | DNA damage, DNA repair, Transcription, Transcription regulation | 2.42 |
| P29692 | EEF1D | Elongation factor 1-delta | 31121.84/4.90 | Stimulate the exchange of GDP bound to EF-1-alpha to GTP | 2.44 |
| O43488 | AKR7A2 | Aflatoxin B1 aldehyde reductase member 2 | 39588.98/6.70 | Produce the neuromodulator gamma-hydroxybutyrate | 2.46 |
| P18085 | ARF4 | ADP-ribosylation factor 4 | 20510.78/6.60 | ADP-ribosyltransferase | 2.73 |
| P52565 | ARHGDIA | Rho GDP-dissociation inhibitor 1 | 23201.77/5.01 | Regulates the GDP/GTP exchange reaction of the Rho proteins | 2.78 |
| O43617 | TRAPPC3 | Trafficking protein particle complex subunit 3 | 20274.10/4.85 | ER-Golgi transport | 2.79 |
| P67775 | PPP2CA | Serine/threonine-protein phosphatase 2A catalytic subunit alpha isoform | 35594.19/ 5.30 | Phosphoprotein phosphatase activity | 2.84 |
| O75323 | GBAS | Protein NipSnap homolog 2 | 33742.64/9.42 | Protein binding | 2.87 |
| P22234 | PAICS | Multifunctional protein ADE2 | 47079.22/6.94 | Purine biosynthesis | 2.95 |
| Q99715 | COL12A1 | Collagen alpha-1(XII) chain | 333146.77 /5.38 | Cell adhesion | 2.95 |
| Q15717 | ELAVL1 | ELAV-like protein 1 | 36091.88/9.23 | 3'-UTR-mediated mRNA stabilization; multicellular organismal development | 3.02 |
| P53004 | BLVRA | Biliverdin reductase A | 33428.49 /6.06 | Reduces the gamma-methene bridge of the open tetrapyrrole, biliverdin IX alpha | 3.05 |
| P11413 | G6PD | Glucose-6-phosphate 1-dehydrogenase | 59256.75/ 6.39 | Carbohydrate metabolism; Glucose metabolism | 3.06 |
| Q12860 | CNTN1 | Contactin-1 | 113320.41/5.62 | Mediate cell surface interactions during nervous system development | 3.09 |
| P62847 | RPS24 | 40S ribosomal protein S24 | 15423.19/10.79 | Structural constituent of ribosome | 3.16 |
| Q9Y2Q0 | ATP8A1 | Phospholipid-transporting ATPase IA | 131369.38/6.41 | Act as aminophospholipid translocase at the plasma membrane in neuronal cells | 3.21 |
| P46777 | RPL5 | 60S ribosomal protein L5 | 34362.62/ 9.73 | Structural constituent of ribosome | 3.43 |
| Q92752 | TNR | Tenascin-R | 149561.96/4.71 | Cell adhesion | 4.3 |
| P04179 | SOD2 | Superoxide dismutase [Mn], mitochondrial | 24722.09/8.35 | Oxidoreductase | 4.35 |
| Q9NYU2 | UGGT1 | UDP-glucose:glycoprotein glucosyltransferase 1 | 177189.59/5.42 | Protein modification; protein glycosylation | 4.77 |
| P07954 | FH | Fumarate hydratase, mitochondrial | 54636.99/8.85 | Tricarboxylic acid cycle, Also acts as a tumor suppressor. | 5.38 |
| Q92823 | NRCAM | Neuronal cell adhesion molecule | 143889.73/5.45 | Neurite outgrowth, cell-cell contacts between Schwann cells and axons, neuron migration | 6.01 |
| P62851 | RPS25 | 40S ribosomal protein S25 | 13742.13/10.12 | Structural constituent of ribosome | 8.87 |
| Q9JKK7 | TMOD2 | Tropomodulin-2 | 39510.83/5.28 | Block the elongation and depolymerization of the actin filaments at the pointed end | 11.32 |
